# Editorial: Fatigue and Recovery in Football

**DOI:** 10.3390/sports7080192

**Published:** 2019-08-13

**Authors:** Neil Clarke, Mark Noon

**Affiliations:** School of Life Sciences, Coventry University, Coventry CV1 5FB, UK

The football codes (soccer, American football, Australian rules football, rugby league, and union and Gaelic football) are intermittent team sports with bouts of high-intensity activity interspersed with low-intensity activities or rest. A range of stresses placed upon intermittent team sport athletes result in transient, acute and chronic fatigue. Fatigue is complex and multifactorial, and is dependent on many contextual factors, such as physical capacity, technical qualities, playing position, tactical role, training load, importance of the game and seasonal period. Furthermore, the number of competitive matches per season is often very high; consequently, athletes only have a limited timeframe to recover following training sessions and competition. There is evidence that too many matches can lead to a lack of motivation and mental burn out, as well as a decrease in physical and match performance and an increase in injuries. Therefore, recovery strategies are required to alleviate fatigue, regain performance and reduce the risk of injury. 

The present special issue of *Sports* intends to provide new insights into fatigue and recovery in all football codes. Through acquiring this knowledge, it becomes possible to facilitate superior methods of player management and to help practitioners establish efficient recovery protocols. As an outcome, nine high-quality contributions authored by international experts from Australia, Germany, Ireland, Israel, New Zealand, Singapore, Spain, Qatar and the United Kingdom were published. All contributions consider fatigue and recovery considering the most updated methods and advanced solutions emerging from different fields of expertise.

The opening paper by Barrett et al. [1] undertook a preliminary investigation into the effects of playing position and contextual factors on elite soccer players’ internal match loads, as measured via differential ratings of perceived exertion (dRPE). Positional differences were observed during soccer in players across breathlessness (RPE-B), leg muscle exertion (RPE-L), and technical demand (RPE-T), with full backs reporting substantially higher dRPE than any other position. Players reported higher RPE-T when playing teams at the top of the league. Taken together, these findings show potential for dRPE data collection as a practical method of monitoring internal load during elite soccer match play. 

Next, Meckel et al. [2] investigated the seasonal variations in fitness and performance indices of professional male soccer players. The findings of the study demonstrated significant improvement in the vertical jump, the 4 × 10 m test and flexibility during the preseason, with no further change in the mid-season. Similarly, the indices of aerobic fitness, VO_2max_ and anaerobic threshold (AT), were significantly improved during the preseason, with further significant improvement in AT in mid-season compared to post-preseason measures. However, repeated sprint test indices showed that while the performance decrement was improved during the preseason period, ideal sprint and total times became significantly slower during the preseason period, suggesting possible fatigue symptoms due to the high training volume during the preseason phase.

In a similar vein, Winder et al. [3] used GPS-derived data to assess the impact of playing a competitive match requiring extra time on a competitive 90-min match played 64 h later and elucidated the influence of extra time on post-match recovery. The results demonstrated that there is compromised recovery 48 h following a match requiring extra time in some individual players. Furthermore, performance and recovery were potentially exacerbated during, and following, a match played 64 h later. From a practical perspective, this data suggests 120 min of match play causes deleterious effects on recovery and subsequent performance (in certain players), creating implications for the use of recovery modalities and training prescription following a match requiring extra time.

Becker et al. [4] examined the influence of a fatigued core musculature on the acceleration of the head during jump headers and run headers. This study showed that fatigued core muscles influence the execution of headers in soccer. In contrast to expectations postulated in literature that the acceleration of the head should increase due to a reduced head–neck–torso alignment, the acceleration of the head actually decreases after fatigue. However, to achieve evidence-based practical applications, further investigations are necessary. So far, the assumption is that additional strengthening of the neck flexors and extensors, which is not common in soccer, might play an important role in protecting the head from high impacts during headers in soccer.

Subsequently, Tang et al. [5] investigated the influence that different frequencies of deceleration and acceleration actions had on the physiological demands in professional soccer players. This study represents a first attempt to show that frequent directional changes (decelerations and accelerations) performed at a high intensity running speed alters the “movement demands” and increases the cardiovascular and perceived exertion in professional soccer players. These findings suggest that such deceleration and acceleration actions carry the potential to increase player fatigue and, therefore, should be carefully considered when quantifying the physical performance in team sports such as soccer.

In a change of sport, Swinbourne et al. [6] examined the efficacy of sleep extension in professional rugby players. The findings suggest that professional rugby players are at risk of poor sleep during pre-season training, with concomitant rises in physical stress. However, a sleep extension intervention resulted in athletes improving total sleep time and quality, with beneficial changes in stress hormone expression and reaction time performance compared to a control. As a consequence, implementing a sleep extension programme among professional athletes is recommended to improve sleep, with beneficial changes in stress hormone expression and reaction time performance.

Akubat et al. [7] examined the relationship of external:internal training load ratios with fitness and assessed the impact of fatigue. The results demonstrated that in rested conditions all of the external:internal training load ratios showed large relationships with measures of fitness. However, under conditions where players had not fully recovered there were moderate changes in some ratios and relationships with fitness became weaker. Therefore, the use of some ratios in scenarios where players are not fully recovered should be treated with caution, but also opens an avenue for these ratios to be potentially considered as detectors of fatigue.

Noon et al. [8] determined the sensitivity of selected subjective (e.g., motivation, recovery and sleep quality) and objective (e.g. countermovement jump) monitoring assessments in detecting changes in group and individual responses to low and high load bouts of high-intensity intermittent exercise. The findings suggest that Selected WQ items detected group and individual responses to high load and low load highlighting their potential utility. However, objective assessments lacked the sensitivity to detect small individual changes. As a consequence, selected well-being questionnaire items could provide important information on the recovery status of a player given their sensitivity to changes in high load and low load. Given the time cost of data collection and analysis and the lack of sensitivity to individual changes, daily monitoring using objective assessments may not practical be in team sports. Therefore, daily subjective assessments may provide greater utility in an applied setting compared with objective assessments.

Finally, the study by De Silva et al. [9] is the first to investigate the differences in activity demands during training and competitive matches in relation to playing positions in soccer at an elite soccer academy in order to understand differences between training and match sessions, with respect to different playing positions. The results indicate that, while there are significant position-specific differences in activity levels during matches, such differences are not observed for data pertaining to the training sessions. While causality cannot be inferred from these results, it does open the possibility of designing future investigations to establish the relationship between training and match activity. Furthermore, patterns in position-specific seasonal variations can lead to tailor-made training programmes to cater to different positions of play.

The editors would like to express their sincere gratitude to the authors, who generously shared their scientific knowledge and experience through their contributions; to the peer reviewers, whose contributions significantly enhanced the quality of this Special Issue; and to the managing editors of Sports, who enthusiastically supported and promoted this initiative.
